# Diagnosed Diabetes Prevalence and Risk Factor Rankings, by State, 2014–2016: A Ring Map Visualization

**DOI:** 10.5888/pcd16.180470

**Published:** 2019-04-11

**Authors:** Ana Lòpez-DeFede, John E. Stewart

**Affiliations:** 1 Institute for Families in Society, University of South Carolina, Columbia, South Carolina

**Figure:**
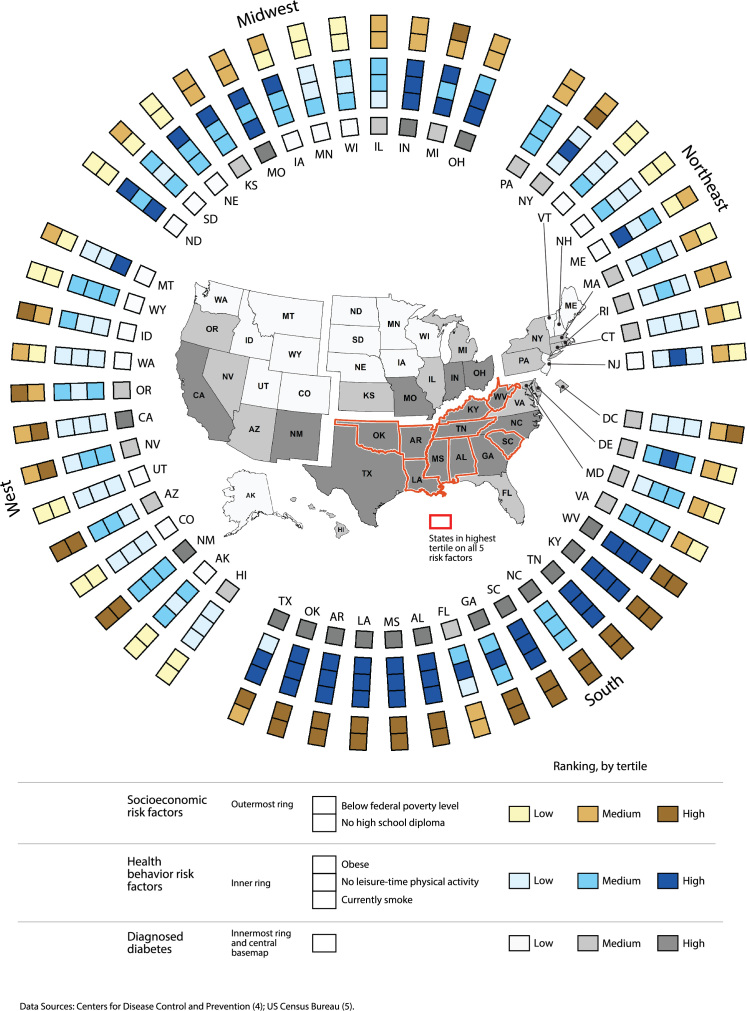
The ring map shows that states with a higher prevalence of risk factors generally have a higher prevalence of diagnosed diabetes. The 9 states in the highest tertile for all 5 risk factors also are in the highest tertile for diabetes prevalence. By integrating multiple spatial data elements in a single graphic, the ring map highlights possible state-level associations between diagnosed diabetes prevalence, socioeconomic disadvantage, and health behaviors. All mapped values represent data for adults aged ≥18, except the percentage with no high school diploma, which was measured for adults aged ≥25.

**Table:** 

State^a^	Prevalence of Diagnosed Diabetes	Socioeconomic Risk Factors		Health Behavior Risk Factors		
		Below Poverty	No High School Diploma	Obese	No Leisure-Time Physical Activity	Currently Smoke
Alabama	High	High	High	High	High	High
Alaska	Low	Low	Low	Medium	Low	Medium
Arizona	Medium	High	High	Medium	Medium	Low
Arkansas	High	High	High	High	High	High
California	High	Medium	High	Low	Low	Low
Colorado	Low	Low	Low	Low	Low	Low
Connecticut	Medium	Low	Medium	Low	Low	Low
Delaware	Medium	Low	Medium	Medium	High	Medium
District of Columbia	Medium	High	Medium	Low	Low	Low
Florida	Medium	Medium	Medium	Low	High	Medium
Georgia	High	High	High	Medium	High	Medium
Hawaii	Medium	Low	Low	Low	Low	Low
Idaho	Low	High	Medium	Medium	Low	Low
Illinois	Medium	Medium	Medium	Medium	Medium	Low
Indiana	High	Medium	Medium	High	High	High
Iowa	Low	Medium	Low	High	Medium	Medium
Kansas	Medium	Medium	Medium	High	Medium	Medium
Kentucky	High	High	High	High	High	High
Louisiana	High	High	High	High	High	High
Maine	Low	Medium	Low	Medium	Low	High
Maryland	Medium	Low	Medium	Medium	Medium	Low
Massachusetts	Medium	Low	Medium	Low	Medium	Low
Michigan	Medium	High	Medium	High	Medium	High
Minnesota	Low	Low	Low	Low	Low	Medium
Mississippi	High	High	High	High	High	High
Missouri	High	Medium	Medium	High	Medium	High
Montana	Low	Medium	Low	Low	Low	High
Nebraska	Low	Low	Low	High	Medium	Medium
Nevada	Medium	Medium	High	Low	Medium	Medium
New Hampshire	Low	Low	Low	Low	Low	Medium
New Jersey	Low	Low	Medium	Low	High	Low
New Mexico	High	High	High	Medium	Medium	Medium
New York	Medium	Medium	High	Low	High	Low
North Carolina	High	High	High	Medium	Medium	Medium
North Dakota	Low	Low	Low	High	Medium	High
Ohio	High	Medium	Medium	Medium	High	High
Oklahoma	High	High	High	High	High	High
Oregon	Medium	High	Medium	Medium	Low	Medium
Pennsylvania	Medium	Medium	Medium	Medium	Medium	Medium
Rhode Island	Medium	Medium	Medium	Low	Medium	Low
South Carolina	High	High	High	High	High	High
South Dakota	Low	Medium	Low	Medium	Low	Medium
Tennessee	High	High	High	High	High	High
Texas	High	Medium	High	High	High	Low
Utah	Low	Low	Low	Low	Low	Low
Vermont	Low	Low	Low	Low	Low	Medium
Virginia	Medium	Low	Medium	Medium	Medium	Medium
Washington	Low	Medium	Low	Low	Low	Low
West Virginia	High	High	High	High	High	High
Wisconsin	Low	Low	Low	Medium	Low	Medium
Wyoming	Low	Low	Low	Medium	Medium	Medium

^a^ The following states were in the highest tertile for all 5 risk factors: Alabama, Arkansas, Kentucky, Louisiana, Mississippi, Oklahoma, South Carolina, Tennessee, and West Virginia.

## Background

In the United States, diabetes is a leading cause of adult-onset blindness, kidney failure, and death (1). Efforts to prevent and control diabetes must consider geographic variation in disease prevalence and risk factors such as obesity, sedentary lifestyle, and low educational attainment (2). Maps are essential to our understanding of geographic differences in population health and disease vulnerability. Comparing geographic patterns of disease and population risk across multiple maps, however, can be cumbersome. Ring mapping is an innovative geovisualization method that permits the display of multiple spatially referenced variables on a single map (3). We used a ring map to depict the prevalence of diagnosed diabetes and 5 associated risk factors (living below the federal poverty level, low educational attainment, obesity, no leisure-time physical activity, and current smoking) for adults in all 50 US states and the District of Columbia.

## Data Sources and Map Logistics

We obtained data on the age-adjusted prevalence of diagnosed diabetes, obesity, physical activity, and current smoking among adults aged 18 or older from the Behavioral Risk Factor Surveillance System (4). For these measures, we calculated mean age-adjusted prevalence on the basis of the most recent 3 years of data available (2014–2016). We obtained data on poverty (percentage of adults aged ≥18 below the federal poverty level) and educational attainment (percentage of adults ≥25 with no high school diploma) from the US Census Bureau, American Community Survey, 2015 1-Year Estimates (5).

We constructed a ring map with 2 principal parts: a ring display and a central basemap. The ring display consists of 6 concentric rings, each comprising 51 symbolization units, 1 unit in each ring for each state and the District of Columbia. The 2 outermost rings represent the 2 socioeconomic risk factors; the 3 inner rings, the 3 health behavior risk factors; and the single innermost ring, the prevalence of diagnosed diabetes. The central basemap shows the geographic pattern of diagnosed diabetes prevalence across states; the shade used to depict the prevalence of diagnosed diabetes in each state on the basemap is the same shade used in the innermost ring. Diabetes and risk factor data are symbolized by using a tertile ranking scheme, with approximately equal numbers of observations in low, medium, and high classes. Tertiles were based on the distribution of values for all 50 states and the District of Columbia (Table). Intentional gaps in the rings and basemap indicate the 4 US Census regions, facilitating exploration of potential regional differences in diabetes prevalence and population risk.

**Table:** Ranges for Low, Medium, and High Tertiles for Prevalence of Diagnosed Diabetes and Selected Associated Risk Factors, Based on Distribution of Values Among Adults Aged ≥18 in 50 States and the District of Columbia^a^

Measure	Low	Medium	High
**Diagnosed diabetes, %**	6.5–8.2	8.3–9.7	9.8–12.8
**Socioeconomic risk factors**			
Below federal poverty level, %	7.6–11.1	11.2–13.8	13.9–18.9
No high school diploma, %^b^	6.4–9.2	9.3–12.4	12.5–17.8
**Health behavior risk factors**			
Obese, %	21.2–27.3	27.4–30.9	31.0–36.8
No leisure-time physical activity, %	16.6–21.3	21.4–24.9	25.0–32.4
Currently smoke, %	9.1–16.0	16.1–19.7	19.8–27.3

a Data sources: Behavioral Risk Factor Surveillance System (4), US Census Bureau, American Community Survey, 2015 1-Year Estimates (5).

b Among adults aged ≥25.

A state-specific example (Montana) illustrates how to interpret the ring map. The ring display shows 6 symbolization units for Montana. Reading from the outermost rings to innermost ring, we see that Montana has a medium prevalence of poverty, a low prevalence of no high school diploma, a low prevalence of obesity, a low prevalence of no leisure-time physical activity, a high prevalence of current smoking, and low prevalence of diagnosed diabetes. The basemap shows the location of Montana and its low prevalence of diagnosed diabetes in relation to the rest of the United States.

The US basemap was created in ArcMap version 10.4 (Esri). A JavaScript was developed to draw the ring elements in Adobe Illustrator (Adobe, Inc). We assembled the basemap and rings and added text and legend elements in Adobe Illustrator.

## Highlights

The ring map shows generally a higher prevalence of diagnosed diabetes in the South. This finding is consistent with the findings of previous research, which identified a “diabetes belt” of counties located predominantly in the South census region (2). The prevalence of socioeconomic and health behavior risk factors is also higher overall in the South. The 9 states in the highest tertile for all 5 risk factors (all located in the South) are also in the highest tertile for diagnosed diabetes. Conversely, of the 3 states in the lowest tertile for all 5 risk factors (all located in the West), 2 states (Colorado and Utah) are in the lowest tertile for diagnosed diabetes and 1 state (Hawaii) is in the medium class.

Some clear exceptions to the general spatial correspondence of diagnosed diabetes prevalence and population risk merit examination. In the midwestern states of Iowa, Nebraska, and North Dakota, for example, obesity prevalence is high, but diabetes prevalence is low. On the other hand, California, in the West, has a low prevalence of obesity, a low prevalence of no leisure-time physical activity, and a low prevalence of smoking but a high prevalence of diagnosed diabetes. Thus, although the ring map highlights possible associations between diagnosed diabetes prevalence, socioeconomic disadvantage, and health behaviors at the state level, it also suggests potential regional differences in risk (6).

This ring map has several limitations. The geovisualization does not indicate the significance of potential associations between the selected risk factors and diabetes prevalence, nor does it convey statistical information about spatial autocorrelation of risk factors and diabetes. Based on state-level data, the ring map does not permit visual assessment of small-area geographic variation in diabetes and population risk within states. Finally, graphic space and legibility constraints limit the number of rings displayed and thus the number of potential risk factors mapped.

## Action

This novel geovisualization can help raise public awareness about spatial variability in diabetes prevalence and vulnerability. The striking visual association between the prevalence of diagnosed diabetes and population risk, especially in the South, can inform and motivate state initiatives to address such modifiable risk factors as poverty, obesity, sedentary lifestyle, and smoking. The ring map also might encourage further exploration of additional area-level factors that alone, or in combination, influence diabetes morbidity and mortality, including, racial/ethnic composition (1,7), characteristics of the built environment (3), and state decisions to expand Medicaid (8).
